# Regional initiatives for malaria elimination: Building and maintaining partnerships

**DOI:** 10.1371/journal.pmed.1002401

**Published:** 2017-10-05

**Authors:** Andrew A. Lover, Kelly E. Harvard, Alistair E. Lindawson, Cara Smith Gueye, Rima Shretta, Roly Gosling, Richard Feachem

**Affiliations:** Malaria Elimination Initiative, Global Health Group at the University of California, San Francisco, San Francisco, California, United States of America

## Abstract

Andrew Lover and colleagues discuss regional malaria initiatives, the strengths and challenges.

**Summary points**

- Country programs and international donors are increasingly focused on regional approaches to malaria elimination; regional initiatives have been established in southern Africa, the Arabian Peninsula, Asia Pacific, Eastern Europe, and Latin America. Despite this growing attention, there is limited information and guidance on both key activities and organizational components of regional initiatives.
- In a review conducted for this policy forum, common characteristics across existing regional initiatives emerged: building a region-specific evidence base, leveraging expertise and resources, shifting commodities or pooling procurement, developing data-sharing systems, mobilizing resources, promoting high-level accountability, and strengthening advocacy.
- Regional initiatives share key structural elements: a strategic unit, technical forums, mechanisms to distribute financing, a high-level political body, and a regional champion or envoy.
- Monitoring and evaluating the impact of regional initiatives is weak, and reporting of funding is very limited. A suitable set of indicators to better evaluate the impact of regional initiatives is needed. Finally, the established aid architecture should adapt to improve proposal and grant management processes for regional initiatives in comparison to national and bilateral grants.

## Why are regional approaches needed?

Well-coordinated efforts are needed to prevent the flow of malaria parasites across international borders, to find solutions for tackling malaria in the final stages of malaria elimination, and to ensure political and financial support once malaria becomes a comparatively rare disease [1]. Regional approaches to malaria elimination and subsequent eradication provide unique solutions to these key challenges (Box 1).

**Box 1. Key issues that regional initiatives (RIs) seek to address.**

Malaria eradication (the permanent cessation of malaria transmission globally) presents new challenges for malaria control programs. These include

- region-specific technical problems in the final stages of elimination;
- movement of people and parasites across international borders potentially impacting both national health and human security;
- decreasing prioritization of malaria within ministries of health due to low caseloads and competing disease priorities leading to limited financing to support malaria elimination; and
- increasingly hidden malaria burden, generally confined to remote regions and marginalized populations.

Regional initiatives (RIs), defined here as multicountry and multisector partnerships with governmental support that work towards malaria elimination, are not novel. The WHO-led Global Malaria Eradication Programme (GMEP; 1959–1970) highlighted 3 benefits from international collaboration: regular data sharing and coordination, special border zones for intensified activities, and administrative systems for health personnel from each country to readily transit borders [2]. While the GMEP did not achieve its stated goal of malaria eradication, its structures were successful in greatly decreasing malaria burden in many regions [2,3]. More recently, the WHO *Global Technical Strategy for Malaria 2016–2030* highlights a need to “deepen regional collaboration” towards elimination [4], as evidenced by modeling studies that confirm that malaria elimination will only be possible in countries like South Africa through collaboration with their higher-endemic neighbors, such as Mozambique, in order to target parasite sources [5].

However, there is no guidance on why RIs are needed for malaria control and elimination and what makes an initiative successful. In this policy forum we review the activities and organizational elements of existing RIs, assess shared challenges, and suggest changes to allow for better incorporation of RIs into existing funding structures. To evaluate these issues, a semi-systematic review of publicly available reports was performed using Google Scholar and PubMed [search strategy: “regional + malaria + (program OR programme)”] and on applicable RI websites to identify RIs (Table 1). As many reports were unindexed, the “ancestor method” of tracing via bibliographies was also used. Bilateral “cross-border” partnerships were not included as the scope of these geographically limited structures is distinct from RIs. Framing questions for this abstraction were as follows:

1. What lessons can be drawn from current and historical malaria regional partnerships?
2. What elements are critical for successful implementation for outcomes?
3. What changes would promote the formation of new partnerships?

**Table 1:** Identified RIs for malaria elimination.

Initiative	Background	Website URL
ALMA	ALMA is a coalition of 49 African heads of state and government working to eliminate malaria by 2030.	http://alma2030.org
AMI and RAVREDA	AMI is a regional program implemented in 11 Amazon Basin and Central American countries that are also members of the RAVREDA and includes ministries of health and technical partners working together to support regional malaria progress.	http://www.usaidami.org
APLMA	APLMA is an affiliation of 22 Asian and Pacific heads of government formed to accelerate progress against malaria and to eliminate it in the entire region by 2030.	http://aplma.org
APMEN	APMEN is a network of 18 national malaria control programs and institutional partners in the Asia Pacific region committed to working towards malaria elimination.	http://apmen.org
Malaria-free Arabian Peninsula Initiative	The Malaria-free Arabian Peninsula Initiative is a consortium of 6 countries based within the GCC working collaboratively to eliminate malaria on the Arabian Peninsula, with a particular focus on highly endemic Yemen and the Yemeni–Saudi Arabian border.	http://sgh.org.sa/en-us/technicalprograms/infictiousdiseases/initiativearabianpeninsulafreeofmalaria.aspx
E8 Regional Initiative	E8 is a coordinated, 8-country effort to achieve the goal of eliminating malaria in 4 southern Africa countries by 2020 (Botswana, Namibia, South Africa, and Swaziland) and to subsequently pave the way for elimination in 4 more by 2030 (Angola, Mozambique, Zambia, and Zimbabwe).	http://malariaelimination8.org/
EMMIE	EMMIE is a consortium of 9 countries and technical partners aiming to achieve malaria elimination in the Mesoamerican subregion and on the island of Hispaniola by 2020. The RI utilizes a cash-on-delivery model to promote and incentivize an accelerated approach to malaria elimination.	http://www.theglobalfund.org/en/portfolio/applicant/?loc=QRA&k=564e7944-7380-4c21-aa31-893ec3429dcf
Mekong Malaria Elimination Hub (formerly ERAR)	ERAR was a coordinated 6-state effort across the GMS, including Yunnan, to immediately respond to and contain drug-resistant *Plasmodium falciparum* malaria. The new regional hub supports efforts to eliminate *P. falciparum* malaria from the GMS by 2025, and all species of human malaria by 2030, in collaboration with RAI.	http://www.who.int/malaria/areas/greater_mekong/en/
MOSASWA (formerly LSDI)	MOSASWA is a trilateral partnership between Mozambique, South Africa, and Swaziland that aims to achieve zero local transmission in Swaziland, South Africa, and Maputo province by 2020 and pre-elimination status in the remainder of southern Mozambique by 2025.	n/a
RAI	RAI is a coordinated 5-country partnership that aims to avert the spread of artemisinin resistance and accelerate elimination of *P. falciparum* malaria in the GMS countries (excepting Yunnan), in collaboration with the ERAR.	http://www.raifund.org/

Abbreviations: ALMA, African Leaders Malaria Alliance; AMI, Amazon Malaria Initiative; APLMA, Asia Pacific Leaders Malaria Alliance; APMEN, Asia Pacific Malaria Elimination Network; E8, Elimination 8; EMMIE, Malaria Elimination in Mesoamerica and the Island of Hispaniola; ERAR, Emergency Response to Artemisinin Resistance; GCC, Gulf Cooperation Council; GMS, Greater Mekong Subregion; LSDI, Lubombo Spatial Development Initiative; MOSASWA, Initiative for Malaria Elimination in Southern Mozambique, South Africa and Swaziland; n/a, not available; RAI, Regional Artemisinin-resistance Initiative; RAVREDA, Amazon Network for the Surveillance of Antimalarial Drug Resistance; RI, regional initiative.

## Activities of RIs

Among the 10 RIs reviewed here (Table 1), a number of key activities came to light (Box 2). Most RIs prioritize building a region-specific evidence base to better inform national policies. Some initiatives, such as the Asia Pacific Malaria Elimination Network (APMEN) sponsor operational research based on individual country needs (APMEN has subsequently transitioned into a secretariat within the Asia Pacific Leaders Malaria Alliance (APLMA) with major changes expected in 2017–2018). Others, like the Amazon Malaria Initiative (AMI), partnering with the Amazon Network for the Surveillance of Antimalarial Drug Resistance (RAVREDA), monitor drug efficacy using standardized protocols throughout the Amazon Basin and Central America. These initiatives all focus on providing local answers to local problems; for example, the AMI developed entomological guidance for the unique malaria vectors within the region [6].

**Box 2. Key activities of current malaria RIs.**

Diverse activities have been prioritized by RIs (Table 2), but the most commonly reported were

- building a region-specific evidence base;
- leveraging expertise and resources from neighboring countries;
- shifting commodities to address stock-outs and pooled procurements of commodities, including rapid diagnostic tests (RDTs), artemisinin-based combination therapy (ACT), and insecticide-treated mosquito net (ITNs);
- providing financing for targeted interventions to underserved or marginalized populations;
- designing and implementing data-sharing systems; and
- serving as platforms for advocacy, resource mobilization, and encouragement of high-level accountability, including friendly competition.

**Table 2:** Major reported activities of RIs for malaria elimination.

Initiative	Region	Dates active	Capacity building and knowledge sharing	Region-specific evidence base	Joint procurement of commodities	Data-sharing platform	Advocacy, resource mobilization, and/or accountability	Implementation of funding
AMI/RAVREDA	Amazon Basin & Central America	2001–present	✓	✓	✓	✓^1^	✓	
ALMA	Africa	2009–present	✓		✓		✓	
APLMA	Asia Pacific	2013–present		✓	✓		✓	
APMEN	Asia Pacific	2009–present	✓	✓			✓	
Malaria-free Arabian Peninsula Initiative	Middle East	2007–unknown	✓		✓		✓	✓
E8	Southern Africa	2009–present	✓	✓	✓	✓	✓	✓
EMMIE	Mesoamerica & Hispaniola Island	2013–present	✓	✓			✓	✓
ERAR	GMS	2013–present	✓	✓	✓	✓	✓	
MOSASWA (formerly LSDI)	Southern Africa	2015–present (previously LSDI from 1999–2011)	✓	✓	✓	✓^2^	✓	✓
RAI	GMS	2013–present	✓	✓	✓	✓^3^	✓	✓

All activities referenced here are included in publicly available resources, including proposals, strategic plans, and operational plans. As a result, this information may not, in some cases, fully reflect the activities and approaches as currently implemented.

^1^ Current epidemiological data sharing within AMI is predominantly bilateral; a regional antimalarial stock monitoring system has been institutionalized by AMI.

^2^ MOSASWA supports the implementation of the E8 regional database.

^3^ The RAI supports the implementation of the ERAR regional database.

Abbreviations: ALMA, African Leaders Malaria Alliance; AMI, Amazon Malaria Initiative; APLMA, Asia Pacific Leaders Malaria Alliance; APMEN, Asia Pacific Malaria Elimination Network; E8, Elimination 8; EMMIE, Malaria Elimination in Mesoamerica and the Island of Hispaniola; ERAR, Emergency Response to Artemisinin Resistance; GMS, Greater Mekong Subregion; LSDI, Lubombo Spatial Development Initiative; MOSASWA, Initiative for Malaria Elimination in Southern Mozambique, South Africa and Swaziland; RAI, Regional Artemisinin-resistance Initiative; RAVREDA, Amazon Network for the Surveillance of Antimalarial Drug Resistance; RI, regional initiative.

RIs allow for national programs to access resources from neighboring countries. Many sets of activities were reported; one example is the Malaria-free Arabian Peninsula Initiative, which supported the implementation of cross-border strategies between Saudi Arabia and Yemen [7]. Several other RIs, including APMEN and the Elimination 8 (E8), convene forums for regional priorities and sponsor fellowship programs to build local capacities, especially in areas with programmatic gaps, like entomology [8,9]. This practical cooperation was reported to be an important incentive for many country programs to participate in RIs.

RIs reported shifting commodities to address stock-outs and removing barriers to health services among high-risk populations. The AMI strengthens antimalarial drug supply chains through regional stockpiling, online collection of inventory data, joint procurement of antimalarial drugs for countries with limited requirements, and regional trainings in supply chain strengthening [6]. RIs have also worked to improve access to malaria prevention, diagnosis, and treatment, especially among mobile and migrant populations, and the Regional Artemisinin-resistance Initiative (RAI), the E8, and the Malaria-free Arabian Peninsula Initiative have all worked to implement malaria border posts towards increasing access to malaria services in marginalized populations.

Another component is the implementation of data-sharing platforms. RIs may also maximize use of health surveillance data by providing regular reports on potential regional hotspots and outbreaks. The Mekong Malaria Elimination Hub, formerly the Emergency Response to Artemisinin Resistance (ERAR), is working towards a regional database to improve case-based surveillance within the Greater Mekong Subregion (GMS). While designed to mesh with each county-level system, this effort has been challenging due to differences in both data structures and management of national malaria information systems [10]. The creation of regional databases is extremely time- and labor-intensive, requiring technical expertise and often advocacy to obtain political support. While real-time data sharing is expected to improve targeting of interventions and decrease outbreak response times, there has been limited publicly available evaluation of regional databases.

RIs also serve as platforms for advocacy, encouraging high-level accountability and resource mobilization. One example (albeit a political union and not an operationalized RI) is the Tashkent Agreement [11]. This Eastern European alliance worked at ministerial levels to eliminate malaria through political advocacy and increased national accountability, leading to malaria elimination certification for the entire WHO European region in 2016. Similarity, the African Leaders Malaria Alliance (ALMA), APLMA, and E8 have implemented high-level scorecards that are reviewed by senior officials to assess country progress, highlight challenges, and enhance accountability. However, these reports might only serve to highlight successes, without any comparable mechanism for “pushing” struggling countries to do more.

Collaborating as an RI member may also provide countries access to new sources of funding, especially for countries with limited Global Fund support due to increasing economic status (e.g., South Africa, Botswana, Thailand, Vietnam, and China’s Yunnan Province). RIs also allow investments in areas within countries that may be low domestic priorities but are regionally important. Southern Mozambique, for example, while not an internal priority due to low transmission relative to other areas in the country, is important to reduce regional malaria parasite flows, and benefits from participation in the E8 and the Initiative for Malaria Elimination in Southern Mozambique, South Africa and Swaziland (MOSASWA, formerly Lubombo Spatial Development Initiative [LSDI]) initiatives.

The 4 Global Fund–supported RIs (E8, Malaria Elimination in Mesoamerica and the Island of Hispaniola [EMMIE], MOSASWA, and RAI) directly disburse funds for implementation through several mechanisms. The RAI (initially focused on cross-border support within the GMS) has now expanded its scope and dispenses Global Fund funding for both regional and national malaria program activities. MOSASWA utilizes an innovative financing mechanism that combines funding from the Global Fund and the private sector [12,13]. The Gulf Cooperation Council (GCC), privately financed by Kuwait, Oman, Qatar, Saudi Arabia, and the United Arab Emirates, provided funding and technical support to their highest-burden neighbor, Yemen, toward improving malaria control along its borders as part of the Malaria-free Arabian Peninsula Initiative [14].

## Challenges of RIs

One demonstration of the importance of RIs is in the sharing of epidemiological data and subsequent coordinated responses (Box 3). However, challenges remain in creating regional databases due to sensitivities over data sharing. To address this issue, increasing human resources within programs to support data sharing (as in the E8), increasing financing to help target high-risk groups (as in the RAI and E8), plus providing additional financing for new activities in areas identified as important through a regional database (MOSASWA) may be effective.

**Box 3. Identified challenges for RIs.**

- Complex negotiations over sensitive topics, including data sharing and cross-border access;
- Accountability between member states;
- Productively engaging neighbors with limited progress;
- Articulating and measuring the impact of a regional program

A second challenge is the establishment of reliable accountability mechanisms so countries are held responsible for progress. To address this, some RIs (like EMMIE) have systems for accountability built directly into their funding structures. In EMMIE’s cash-on-delivery model, participating countries are required to meet set milestones in order to receive monetary disbursements [15]. A third challenge is maintaining well-functioning partnerships with higher-endemic neighbors. Active participation by these higher-burden countries is crucial to the elimination efforts of their low-burden neighbors. To overcome this challenge, RIs must undertake substantial diplomatic and strategic advocacy highlighting benefits for all parties. For example, pooling of resources (laboratories, commodities, technical experts, and finances), which may be out of reach to individual countries, could encourage greater participation by higher-burden partners.

The final, and possibly most pressing, challenge is evaluating the impact of existing RIs. Very few RIs have prioritized systematic monitoring and evaluation for outcomes; and where independent evaluations have been undertaken, results may not be public. One exception is the evidence for epidemiological impact of RIs obtained from LSDI (now MOSASWA) in southern Africa. Beginning in 1999, this initiative contributed to sustained declines in malaria incidence, which unfortunately were rapidly undone when funding ended in 2011 [16]. To address this major gap, RIs should be explicitly funded for evaluations that measure their impact, ensuring cost-effective implementation.

## Organizational structure of RIs

RIs must be actively designed and managed to ensure they are well aligned with the partnership’s aims. Once an RI has well-delineated goals, several components should be considered to operationalize the partnership (Table 3).

**Table 3:** Important components of malaria RIs.

Initiative	Strategic core	Technical forum(s)	High-level political body	Regional champion or special envoy
AMI/RAVREDA	✓			
ALMA	✓		✓	✓
APLMA	✓	✓	✓	✓
APMEN	✓	✓		
Malaria-free Arabian Peninsula Initiative			✓	
E8	✓	✓	✓	✓
EMMIE	✓		✓	
ERAR	✓			
MOSASWA (formerly LSDI)	✓	✓	✓	
RAI	✓			

Note: As a subset of E8 countries, MOSASWA works in close conjunction with the E8 Initiative and synchronizes certain structural components with existing E8 processes. This generally includes convening technical meetings and high-level political consultations for MOSASWA-specific programs at E8 functions or within E8 subsidiary bodies.

Abbreviations: ALMA, African Leaders Malaria Alliance; AMI, Amazon Malaria Initiative; APLMA, Asia Pacific Leaders Malaria Alliance; APMEN, Asia Pacific Malaria Elimination Network; E8, Elimination 8; EMMIE, Malaria Elimination in Mesoamerica and the Island of Hispaniola; ERAR, Emergency Response to Artemisinin Resistance; LSDI, Lubombo Spatial Development Initiative; MOSASWA, Initiative for Malaria Elimination in Southern Mozambique, South Africa and Swaziland; RAI, Regional Artemisinin-resistance Initiative; RAVREDA, Amazon Network for the Surveillance of Antimalarial Drug Resistance; RI, regional initiative.

Foremost is a dedicated strategic group for logistics and coordination. This core should be responsible for soliciting feedback on strategy, convening meetings with regular updates, and implementing the RI’s decisions. While a strong leader with broad technical and managerial skills is required, the importance of having a charismatic “human engine” to personally push the program forward cannot be overstated [2,17].

The second component is a technical forum, potentially with technical working groups. These must be geared towards addressing the challenges to elimination that have been prioritized by member states and may benefit from including other regional stakeholders. The members of these forums should have clear decision-making authority to determine research focuses, as well as set the technical priorities of the RI. The E8 and APMEN both use this structure to build regional work plans, as the “hows” of elimination are context-specific and countries are accumulating evidence as they implement.

The third structure is a high-level political body, which may include policy-makers from ministries of health, finance, and other sectors (forestry, mining, etc.). This political unit should have authority to authorize national commitments, advocate to higher levels within governments for malaria elimination, and have access to the tools for tracking progress of member states. RIs should also have a well-defined evidence-to-policy process to rapidly inform national elimination planning. APLMA and ALMA serve these functions and advocate for malaria elimination in their regions by highlighting progress, communicating regional benefits of elimination to decision-makers, and ensuring continued engagement in the elimination agenda.

Finally, programs may consider a regional champion or special envoy. This high-profile advocate becomes increasingly important in elimination settings as morbidity and mortality from malaria become less visible. The E8 currently has Dr. Richard Nchabi Kamwi, the former Minister of Health and Social Services for Namibia, to keep malaria high on national agendas, mobilize regional resources, and help “pull” along neighboring countries with less-developed elimination programs. APLMA utilizes Dr. Nafsiah Mboi, the former Minister of Health for Indonesia, as a special envoy to promote global health diplomacy and increase country-level political engagement within Asia Pacific [18].

## Changes needed

Institutional donors and foundations are generally not well structured to support the development, implementation, or monitoring of regional grants, and challenges with grant-writing materials and project administration have been highlighted before [19]. A review of challenges for RIs across all 3 Global Fund disease areas (HIV, tuberculosis [TB], and malaria) concluded that while there has been some progress in streamlining application materials, important barriers remain in design of grant materials, points of contact, and cooperation between country coordinating mechanisms (CCMs) and national programs [20].

For RIs to be successful, donors must develop grant guidance and materials that are well aligned with the unique characteristics of regional grants. Grant applications for RIs should have sections for explicit consideration of benefits and value added from regional cooperation; justification for the specific organizational structure; and streamlined reporting systems, especially if other malaria grants exist in-country. Other sections should consider the roles of accountability amongst country peers and the structures for support from well-resourced to less-resourced partners.

For maximum efficiencies, large funders should also develop practical plans to reduce the administrative burdens from grant development and management and to align indicators and reporting requirements across multiple grants. Fully aligned with this is the recent announcement that all support from the Global Fund to the GMS will be administered through the RAI as a regional block grant to improve efficiency. Portfolio managers should be well versed in specific regional situations, including infrastructure, bureaucratic systems, human resources within the health sector, and data capture and utilization. Finally, there is need to better measure the benefits and impact of RIs; one possible exercise to improve the definition of “success” might be a cost-effectiveness evaluation of RIs relative to separate funding.

## Limitations

While a comprehensive evidence review was undertaken for this policy forum, it is not without limitations. Many documents were unindexed “grey” literature, and documentation from the 10 identified RIs, or information related to other initiatives, may have been overlooked. The second major gap is a lack of budgetary information for any of the RIs examined—the costs associated with RI incorporation and management has not been reported in publicly available documentation. This gap could hamper budget discussions for new initiatives, and addressing it should be a high priority for all current RIs.

## Conclusions

RIs have an important role to play in the malaria elimination landscape but require careful design, implementation, and evaluation to ensure alignment with outcomes. In particular, efforts must be made to assist new partnerships in setting up impactful and sustainable RIs through the design, financing, and evaluation stages. The current leader in this area is the Global Fund, which is financing several RIs, namely E8, EMMIE, MOSASWA, and RAI. The APLMA/APMEN co-secretariat is funded through the Australian Government and the Bill & Melinda Gates Foundation and is attempting to attract other donors who traditionally fund bilateral partnerships. Funders can lead and support the systematic changes necessary to better incorporate RIs into the existing aid architecture by improving guidelines for grant proposal and grant management processes and by commissioning research on suitable indicators. Impact may be easier to demonstrate where RIs can directly invest in targeted interventions, as compared to impact assessment of RIs focused on advocacy and data sharing. Finally, existing RIs must provide evidence for their successes in coordinating multicountry partnerships for malaria elimination to ensure both their own sustainability and further investments in the future.

## Abbreviations

ACT: artemisinin-based combination therapy
ALMA: African Leaders Malaria Alliance
AMI: Amazon Malaria Initiative
APLMA: Asia Pacific Leaders Malaria Alliance
APMEN: Asia Pacific Malaria Elimination Network
CCM: country coordinating mechanism
E8: Elimination 8
EMMIE: Malaria Elimination in Mesoamerica and the Island of Hispaniola
ERAR: Emergency Response to Artemisinin Resistance
GCC: Gulf Cooperation Council
GMEP: Global Malaria Eradication Programme
GMS: Greater Mekong Subregion
ITN: insecticide-treated mosquito net
LSDI: Lubombo Spatial Development Initiative
MOSASWA: Initiative for Malaria Elimination in Southern Mozambique, South Africa and Swaziland
RAI: Regional Artemisinin-resistance Initiative
RAVREDA: Amazon Network for the Surveillance of Antimalarial Drug Resistance
RDT: rapid diagnostic test
RI: regional initiative
TB: tuberculosis
